# Protective Effect of *Gastrodia elata* Polysaccharide GEP-2 Against Oxidative Stress in Intestinal Epithelial NCM460 Cells

**DOI:** 10.3390/ijms27062655

**Published:** 2026-03-14

**Authors:** Yongjiang Yao, Xingjian Wen, Xuefeng He, Dan Liao, Mengting Li, Jiuyu Fan, Rui Liang, Xiaoqi Huang, Na Li

**Affiliations:** 1 Key Laboratory of Chinese Medicinal Resource from Lingnan (Ministry of Education), School of Pharmaceutical Sciences, Guangzhou University of Chinese Medicine, Guangzhou 510006, China; yjiang0206@163.com; 2 Chongqing Academy of Chinese Materia Medica, Nanshan Road 34, Chongqing 400065, China; wenxingjian1990@163.com (X.W.);; 3 Bioengineering College, Chongqing University, Chongqing 400030, China

**Keywords:** *Gastrodia elata* polysaccharides, oxidative stress, intestinal epithelial cells, JAK/STAT signaling pathway, Nrf2/NQO1 signaling pathway, transcriptomics

## Abstract

Oxidative stress in intestinal epithelial cells has been increasingly recognized as a key factor in various intestinal disorders. *Gastrodia elata* polysaccharide-2 (GEP-2), a water-soluble polysaccharide known for its antioxidant properties, has shown potential against intestinal injury. However, its effects on intestinal epithelial cells and the molecular mechanisms involved are not yet fully understood. In this study, we established a hydrogen peroxide (H_2_O_2_)-induced oxidative stress model using human colonic epithelial cells (NCM460) to evaluate the protective effects of GEP-2. We assessed cell viability, antioxidant enzyme activities, reactive oxygen species (ROS) levels, and mitochondrial membrane potential (MMP). The results demonstrated that GEP-2 pretreatment significantly improved the viability of NCM460 cells subjected to H_2_O_2_ damage. Additionally, it could enhance the antioxidant defense, reduce the levels of ROS, malondialdehyde (MDA), and maintain the MMP. Transcriptomic analysis identified 169 differentially expressed genes upregulated in the glutathione metabolism. JAK-STAT pathway and downregulated in inflammation. Furthermore, it was shown that GEP-2 treatment activated the Nuclear factor erythroid 2-related factor 2 (Nrf2)/quinone oxidoreductase 1 (NQO1)-mediated antioxidant response and promoted the Janus kinase (JAK)/signal transducer and activator of transcription (STAT) signaling pathway. Therefore, GEP-2 exerts multi-targeted cell protection by coordinating the Nrf2/NQO1 antioxidant axis and the JAK/STAT survival signaling pathway, providing a theoretical basis for the development of novel antioxidants.

## 1. Introduction

The intestine serves not only as the main site for the digestion and absorption of nutrients, but also as a critical component of the body’s immune defense system, effectively preventing the invasion of pathogens and harmful compounds [1]. As the primary contact layer between the intestinal lumen and the internal environment, intestinal epithelial cells (IECs) are constantly exposed to various physical and chemical stimuli [2,3], making them highly sensitive targets for oxidative stress. Under normal conditions, intracellular reactive oxygen species (ROS) are maintained at a moderate level and participate in regulating key cell signal transduction pathways [4]. However, once the cell’s antioxidant capacity is insufficient to eliminate the excessive production of ROS, it will lead to an imbalance in the redox system, triggering a series of pathological changes such as lipid peroxidation, protein denaturation, and DNA damage [5]. Studies have shown that, in IECs, chronic oxidative stress can damage the integrity of tight junction structures, induce mitochondrial dysfunction, and activate pro-inflammatory signal cascades, thereby gradually weakening the epithelial barrier function [6,7,8]. This evidence suggests that oxidative stress plays a crucial role in the mechanism of intestinal epithelial structure damage and mucosal microenvironment disorder.

Synthetic antioxidants, including butylated hydroxyanisole (BHA) and butylated hydroxytoluene (BHT), have been extensively utilized owing to their antioxidant efficacy. However, due to concerns about their long-term safety, such as potential liver and kidney toxicity as well as genetic toxicity risks, their wide application has been restricted [9]. Researchers have been conducting in-depth exploration of natural antioxidant components with better biocompatibility. In recent years, active compounds derived from nature have become an important hotspot in the development of functional foods and biomedical research [10,11,12,13]. Among them, polysaccharides derived from traditional Chinese herbal medicines have attracted much attention due to their diverse chemical structures, wide range of action targets, and high clinical application safety. Research evidence shows that these polysaccharides can effectively eliminate free radicals, activate the endogenous antioxidant defense mechanism in cells, and regulate the signaling pathways closely related to cell survival, thereby exerting significant protective effects in alleviating oxidative stress damage in the gastrointestinal tract [14,15].

*Gastrodia elata* Blume, a well-established traditional medicinal herb, has a long-standing application in the treatment of neurological disorders. Recent pharmacological studies have shown that polysaccharides from *Gastrodia elata* Blume, as the main bioactive component of *Gastrodia elata* Blume, possess a variety of biological functions, including significant neuroprotection, immune regulation, and antioxidant effects [16,17,18]. Our previous research indicates that total polysaccharides from *Gastrodia elata* can effectively alleviate intestinal mucosal damage in mice induced by cyclophosphamide (CTX), reduce oxidative stress damage in intestinal tissues, regulate intestinal barrier function, and highlight its potential role in intestinal protection [19,20]. Oxidative stress has been confirmed to be the basic mechanism of cell damage induced by CTX. Excessive reactive oxygen species (ROS) can directly damage cellular macromolecules and ultimately lead to cellular dysfunction or death. More generally, oxidative stress is widely acknowledged as a central mediator of intestinal epithelial injury induced by various exogenous insults [21]. Although the antioxidant properties of *Gastrodia elata* polysaccharides have been documented, their regulatory role in JAK/STAT signaling within intestinal epithelial cells under oxidative stress remains largely unexplored.

Building on previous findings [22], this study established an H_2_O_2_-induced oxidative stress model in NCM460 cells to investigate the protective effects and underlying mechanism of *Gastrodia elata* polysaccharide-2 (GEP-2), a water-soluble low-molecular-weight polysaccharide of ginseng isolated in our previous study [20]. To evaluate the antioxidant properties of GEP-2, we assessed cell viability, mitochondrial membrane potential (MMP), intracellular ROS level, and antioxidant parameters, including total antioxidant capacity (T-AOC), total superoxide dismutase (T-SOD) activity, catalase (CAT) activity, and glutathione (GSH) content. Next, transcriptome sequencing was conducted to identify genes that were differentially expressed in response to GEP-2 treatment. To confirm the involvement of key signaling pathways, particularly those related to nuclear factor erythroid 2-related factor 2 (Nrf2), Janus kinase (JAK), and signal transducer and activator of transcription 3 (STAT3), further validation was carried out using Western blotting and reverse transcription–quantitative polymerase chain reaction (RT-qPCR). Through this methodology, this study aims to explain how GEP-2 alleviates intestinal oxidative stress to offer a mechanistic foundation for the development of *Gastrodia elata* as a natural intestinal protective agent.

## 2. Results

### 2.1. GEP-2 Displays Robust In Vitro Antioxidant Activity

**Figure 1:**
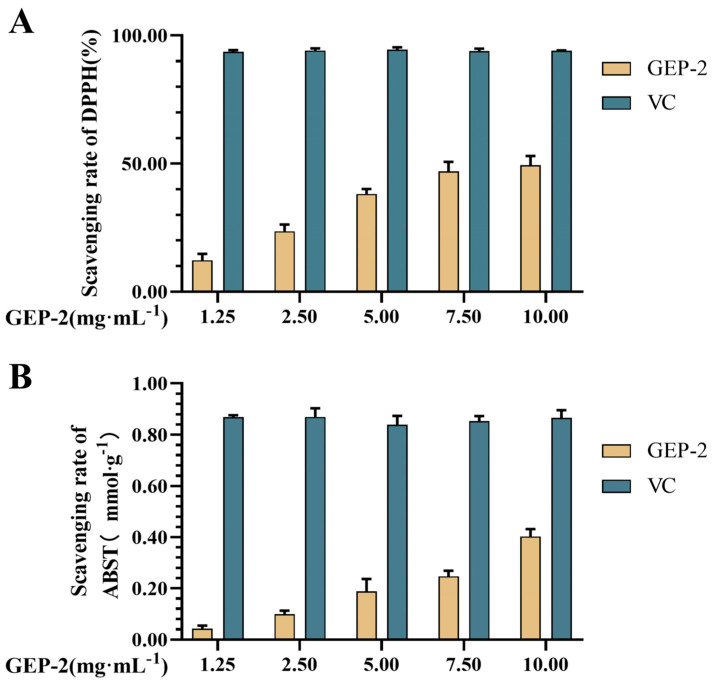
Potential antioxidant activity of GEP-2. (**A**) DPPH and (**B**) ABTS+ cation radical scavenging activities at concentration of 1.25 to 10.0 mg·mL^−1^ (n = 3). VC as a positive control. Values are expressed as mean ± SEM.

As depicted in Figure 1, a comparative assessment of the antioxidant activity between GEP-2 and vitamin C (VC) was carried out via two in vitro assays, namely the DPPH (Figure 1A) and ABTS^+^ (Figure 1B) assays. The outcomes indicate that GEP-2 manifests a concentration-dependent free radical scavenging ability in both systems. At all tested concentrations, GEP-2 demonstrated consistently lower activity compared to the positive control, VC. These findings support the in vitro antioxidant properties of GEP-2, offering a foundation for its potential use as a natural antioxidant.

### 2.2. GEP-2 Mitigates H_2_O_2_-Induced Reduction in the Viability of NCM460 Cells

#### 2.2.1. Establishment of the H_2_O_2_-Induced Model

**Figure 2:**
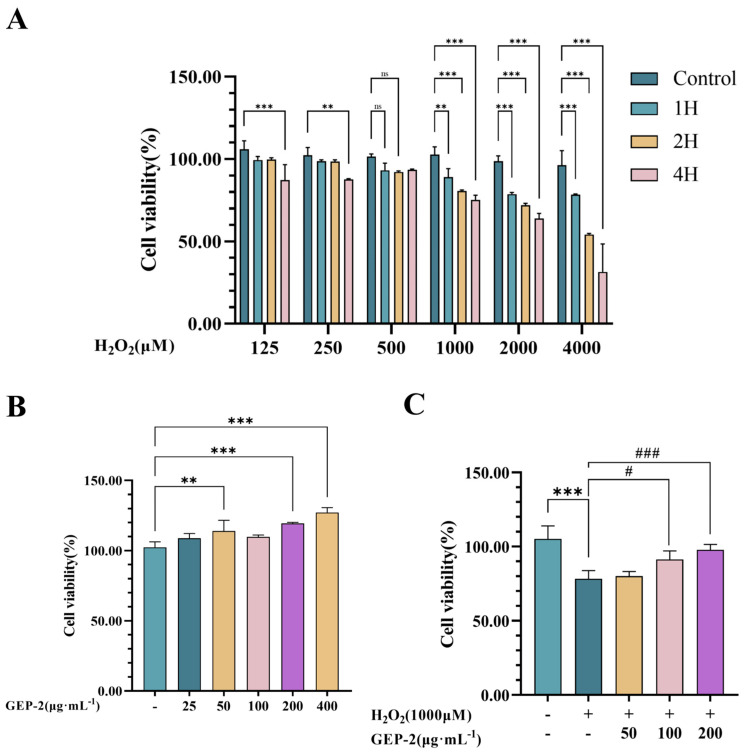
Influence of H_2_O_2_ and GEP-2 on H_2_O_2_-induced cytotoxicity. (**A**) Viability of cells after exposure to different concentrations of H_2_O_2_ (125–4000 μM) for various time intervals (n = 3). (**B**) Viability of cells after exposure to different concentrations of GEP-2 (n = 4). (**C**) Protective efficacy of GEP-2 against H_2_O_2_-induced (1000 μM) cytotoxicity (n = 4). Values are expressed as mean ± SEM. Compared with the Control group: ** *p* < 0.01, *** *p* < 0.001; Compared with the Model group: ^#^ *p* < 0.05, ^###^ *p* < 0.001.

In order to establish a stable in vitro oxidative stress model, the current study utilized different concentrations (250–4000 μM) of H_2_O_2_ to treat NCM460 cells. Samples were collected at various time points (1–4 h) for the analysis of cell viability. As depicted in Figure 2A, the cytotoxic effects of H_2_O_2_ demonstrated both concentration- and time-dependent properties. Exposure to 1000 μM H_2_O_2_ for 4 h led to a reduction in cell viability to 75.16% (*p* < 0.001). This condition satisfied the modeling criteria and was thus determined as the optimal parameter set for establishing the oxidative stress injury model in subsequent experiments.

#### 2.2.2. Safety Evaluation and Experimental Concentration Screening of GEP-2

Figure 2B illustrates that the treatment with GEP-2 at concentrations ranging from 25 to 400 μg·mL^−1^ for 24 h did not exhibit cytotoxicity. GEP-2 significantly enhanced cell viability. These results suggest that GEP-2 has good biosafety within this concentration range. Based on these results, concentrations of 50, 100 and 200 μg·mL^−1^ were chosen for further analysis.

#### 2.2.3. Protective Efficacy of GEP-2 Against H_2_O_2_-Induced Cytotoxicity

Pretreatment with GEP-2 reveals antagonistic effects against H_2_O_2_-induced cellular damage. The results (Figure 2C) show that treatment with 1000 μM H_2_O_2_ significantly decreased cell viability in comparison to the normal group (*p* < 0.001). Conversely, cells pretreated with GEP-2 for 24 h displayed a notable concentration-dependent increase in viability relative to the model group. It confirmed that pretreatment with GEP-2 effectively alleviates the H_2_O_2_-induced reduction in cell viability and exerts a distinct protective effect against oxidative stress injury.

#### 2.2.4. GEP-2 Inhibits H_2_O_2_-Induced Intracellular ROS Overproduction

**Figure 3:**
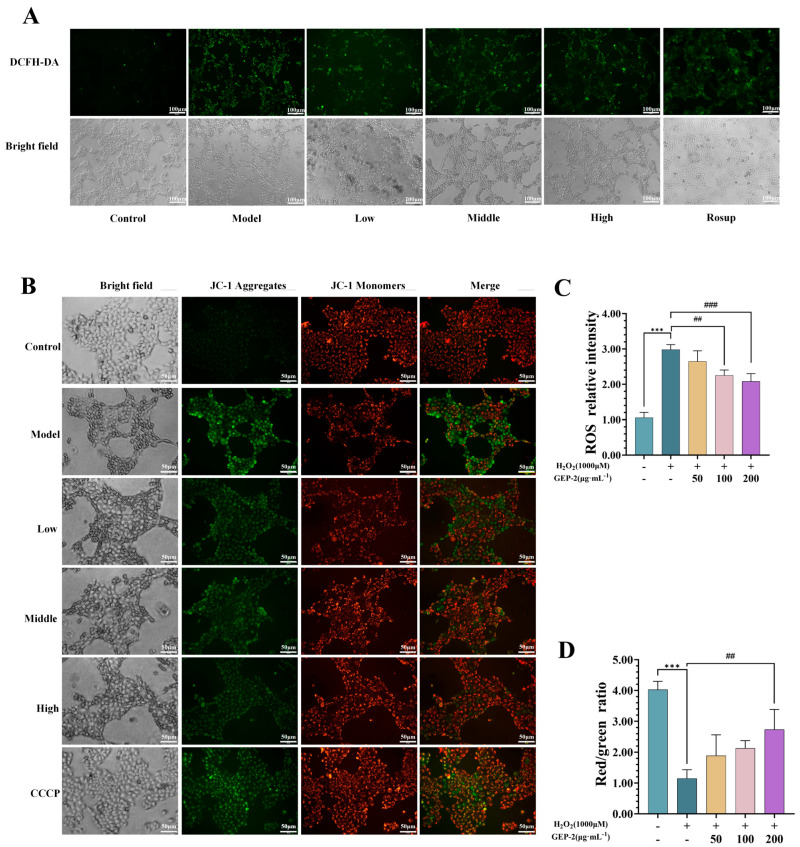
Effect of GEP-2 on Mitochondrial Membrane Potential (MMP) and Reactive Oxygen Species (ROS) in H_2_O_2_-treated NCM460 Cells. (**A**) Representative images depicting ROS accumulation (n = 3). Scale bar: 100 μm. (**B**) Fluorescence images of MMP (n = 3). Scale bar: 50 μm. (**C**) Relative fluorescence intensity of ROS (n = 3). (**D**) The ratio of red to green fluorescence intensity (n = 3). Values are expressed as mean ± SEM. Compared with the Control group: *** *p* < 0.001; Compared with the Model group: ^##^ *p* < 0.01, ^###^ *p* < 0.001.

The fluorescent probe DCFH-DA was employed to assess the antioxidant capacity of GEP-2 by measuring the intracellular ROS level. As shown in Figure 3A,C, in comparison with the unexposed group, the exposed group exhibited a significant increase in green fluorescence intensity (*p* < 0.001), suggesting the accumulation of intracellular ROS. Conversely, pretreatment with GEP-2 led to an obvious reduction in fluorescence intensity. The fluorescence intensity of the high-dose pretreatment group was lower than that of the exposed group (*p* < 0.01). These results suggest that GEP-2 could suppress or curb the H_2_O_2_-triggered excessive generation of ROS, consequently mitigating intracellular oxidative stress and curbing oxidative injury in NCM460 cells.

#### 2.2.5. GEP-2 Relieves H_2_O_2_-Induced Reduction in Mitochondrial Membrane Potential in NCM460 Cells

Excessive accumulation of reactive oxygen species (ROS) can damage mitochondrial function, leading to a decrease in mitochondrial membrane potential (ΔΨm), subsequently impairing mitochondrial function. This is a key factor in oxidative stress-induced cell damage. To further investigate whether GEP-2 helps maintain mitochondrial integrity under oxidative stress conditions, we evaluated the changes in ΔΨm using JC-1 staining. The results are shown in Figure 3B,D. Cells in the normal group mainly showed red fluorescence and maintained normal morphology, indicating intact mitochondrial function. However, cells in the model group showed reduced red fluorescence and increased green fluorescence. Additionally, significant morphological changes were observed, including a decrease in cell number and size, which are consistent with the depolarization of mitochondria and early apoptosis. Pretreatment with GEP-2 improved these indicators, manifested as increased red fluorescence, decreased green fluorescence, and improved cell morphology compared to the exposure group. This indicates that GEP-2 can alleviate H_2_O_2_-induced oxidative stress and help maintain mitochondrial membrane potential.

#### 2.2.6. GEP-2 Alleviates Oxidative Stress by Strengthening the Intrinsic Antioxidant Defense Systems

**Figure 4:**
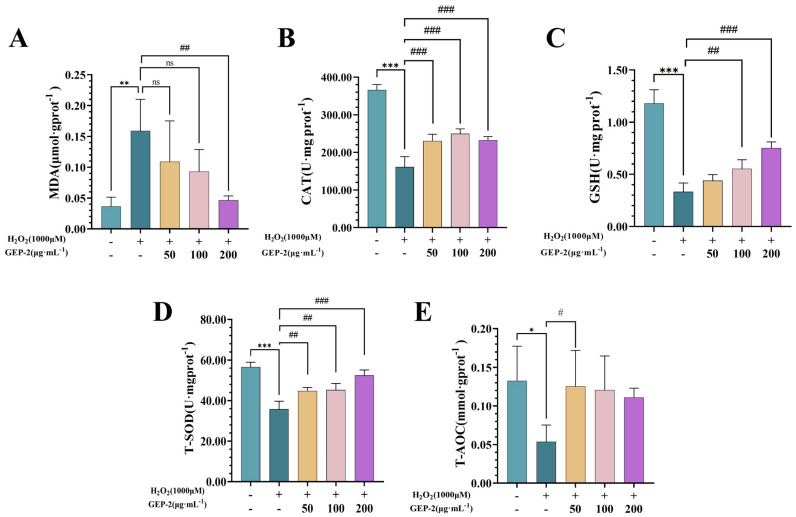
Investigates the antioxidant characteristics of GEP-2 in alleviating H_2_O_2_-induced oxidative stress within cells. (**A**) MDA; (**B**) CAT; (**C**) GSH; (**D**) T-SOD; (**E**) T-AOC. (n = 4). Values are expressed as mean ± SEM. Compared with the Control group: * *p* < 0.05, ** *p* < 0.01, *** *p* < 0.001; Compared with the Model group: ^#^ *p* < 0.05, ^##^ *p* < 0.01, ^###^ *p* < 0.001.

We further evaluated the antioxidant stress effect of GEP-2 by detecting the damage caused by oxidative stress and the parameters related to antioxidant capacity. As shown in Figure 4, the results showed that, after H_2_O_2_ stimulation, the content of MDA in the cells of the model group significantly increased (*p* < 0.001), while the levels of GSH, CAT, T-SOD and T-AOC significantly decreased (*p* < 0.01). These results indicate that H_2_O_2_ treatment effectively induced oxidative stress damage, demonstrating that we successfully constructed a cell oxidative stress damage model.

Following a 24 h pretreatment with GEP-2, the aforementioned oxidative damage and antioxidant imbalance were partially alleviated in a dose-dependent manner. All concentrations of GEP-2 significantly decreased the intracellular MDA content while enhancing the activities of antioxidant enzymes. Notably, medium and high doses of GEP-2 demonstrated remarkable restorative effects on the activities of GSH and T-SOD, as well as the T-AOC, restoring these parameters to levels approaching or even equivalent to those of the normal group.

### 2.3. Transcriptomics-Based Analysis Unveils the Mechanism of GEP-2 in Alleviating Oxidative Stress

#### 2.3.1. Analysis of Differential Gene Expression Among Experimental Groups

**Figure 5:**
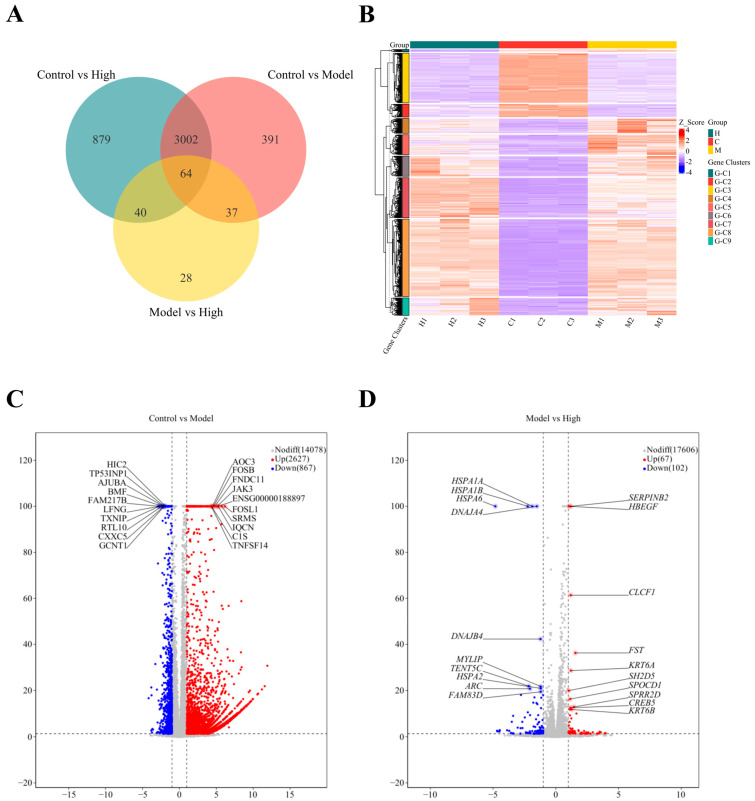
Analysis of Differentially Expressed Genes (DEGs) in NCM460 via Transcriptomics. (**A**) Venn Diagram; (**B**) Hierarchical Clustering Heatmap of DEGs; (**C**) Volcano Plots of DEGs between the control and model groups; (**D**) Volcano plot of DEGs between the model and high-treatment groups.

To explore the molecular mechanisms underlying the protective effects of GEP-2 against oxidative stress, transcriptome sequencing was performed on samples from the Control, Model, and High-dose group. In comparison with the Control group, treatment with H_2_O_2_ led to extensive transcriptional alterations in the untreated group, with a total of 3494 differentially expressed genes (DEGs), comprising 2627 upregulated and 867 downregulated genes (Figure 5A–C). These significant expression changes in gene expression reflect extensive transcriptional reprogramming induced by oxidative stress.

In contrast, high-dose GEP-2 treatment displayed 169 specifically altered DEGs, among which 67 were upregulated and 102 were downregulated (Figure 5D). Venn diagram analysis further clarified the relationships among the three groups (Figure 5A). Although the GEP-2-responsive gene set (High vs. Model) partially overlapped with the injury-associated gene set (Model vs. Control), the two groups of DEGs were largely disparate. This pattern implies that GEP-2 does not globally reverse H_2_O_2_-induced transcriptional changes; rather, it selectively regulates a subset of genes closely related to oxidative stress responses.

#### 2.3.2. Gene Ontology Functional Enrichment Analysis of Differentially Expressed Genes

**Figure 6:**
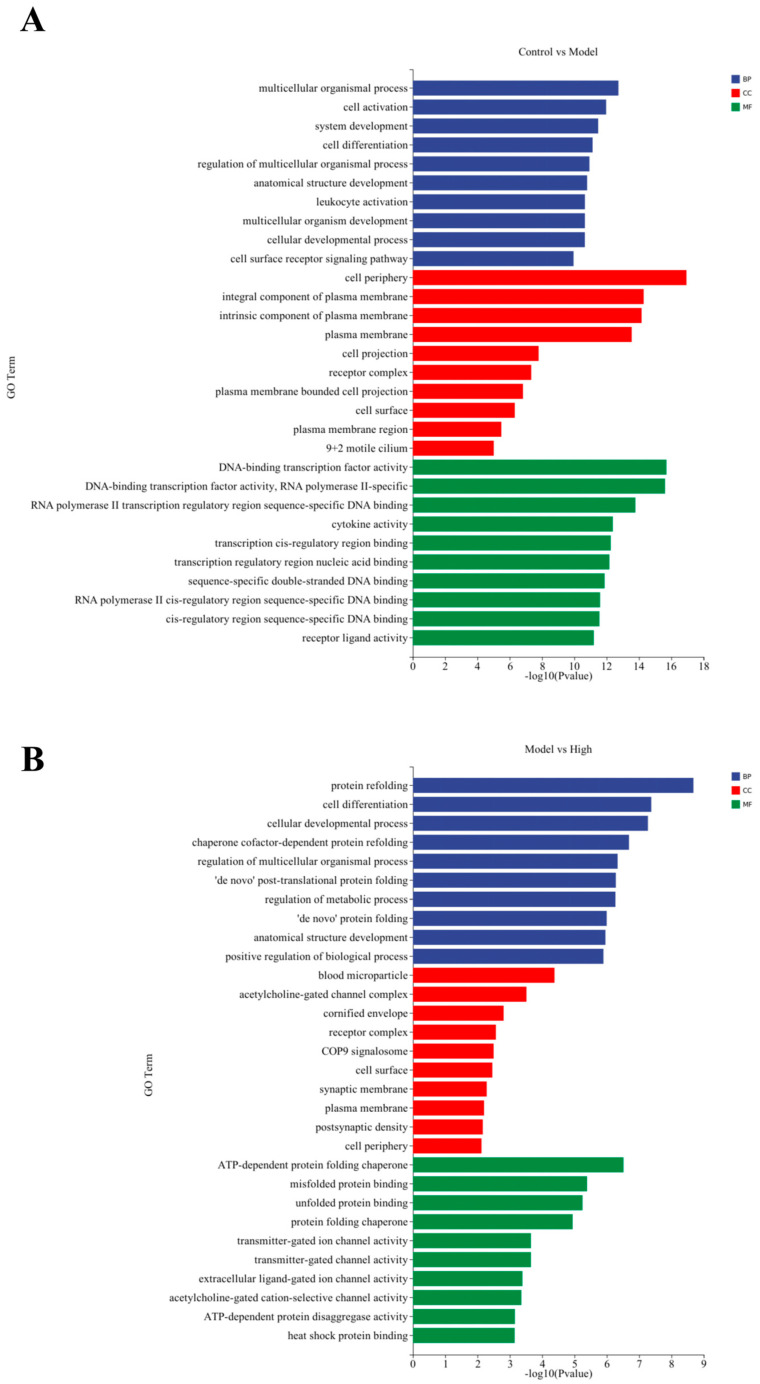
Gene Ontology (GO) term enrichment analysis of different experimental conditions. (**A**) Comparison between the control and model groups. (**B**) Comparison between the model and high-treatment groups.

Compared to the control group, differentially expressed genes in the model group were primarily enriched in immune-related and developmental functions. From the perspective of biological processes (Figure 6A) these genes were significantly associated with cell activation, immune system regulation, and responses to external stimuli. Analysis of cellular components indicated that a considerable proportion of these genes were localized within extracellular regions, plasma membranes, and receptor complexes. At the molecular function level, enrichment was mainly observed in receptor ligand activity, cytokine activity, and signaling receptor activator activity. Collectively, this enrichment pattern reflects a marked immune-inflammatory response and cellular remodeling induced by H_2_O_2_ exposure.

Conversely, the DEGs in the high-dose GEP-2 group were enriched in pathways associated with the maintenance of cellular homeostasis (Figure 6B). Biologically, the prominently enriched categories encompassed responses to unfolded proteins, endoplasmic reticulum stress responses, and the regulation of cellular redox homeostasis. At the cellular component level, the associated genes were primarily localized in the endoplasmic reticulum lumen, endoplasmic reticulum membrane, as well as extracellular exosomes. At the molecular function level, these genes were primarily associated with unfolded protein binding, antioxidant activity, and chaperone functions. This strongly implies that the protective effect of GEP-2 is closely tied to intrinsic adaptive mechanisms, such as regulating endoplasmic reticulum stress, promoting proper protein folding, and enhancing cellular antioxidative capacity.

#### 2.3.3. Transcriptomic KEGG and GSEA Analyses Unveil the Regulatory Function of GEP-2 in Signaling Pathways

**Figure 7:**
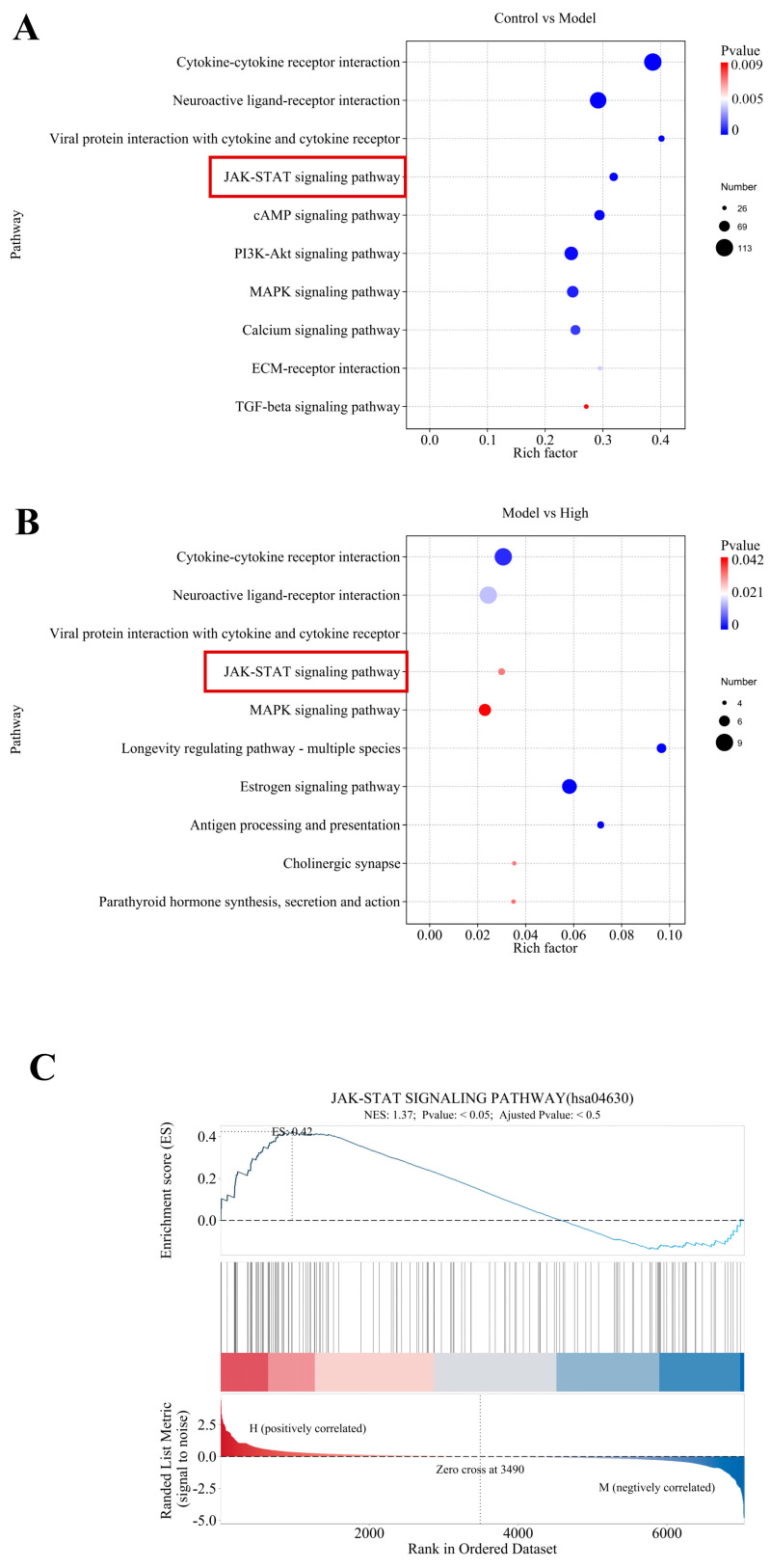
Differentially Expressed Genes (DEGs) were analyzed via Kyoto Encyclopedia of Genes and Genomes pathway enrichment (KEGG) and Gene Set Enrichment Analysis (GSEA). (**A**) KEGG analysis of DEGs between the Control group and the Model group. (**B**) KEGG analysis of DEGs between the Model group and the High-dose group. (**C**) GSEA plots depicting the enrichment of the JAK–STAT signaling pathway (KEGG ID: hsa04630) across different groups.

This research conducted KEGG pathway enrichment analysis on DEGs between the different group to investigate the biological pathways associated with the disease model. The results demonstrate significant enrichment of the JAK–STAT signaling pathway (*p* < 0.05), with an enrichment factor of approximately 0.25, indicating substantial gene participation (Figure 7A).

To assess the influence of high-dose treatment on pathway regulation, KEGG enrichment analysis was carried out by comparing the Model group and the High-dose group. Figure 7B shows that the JAK-STAT signaling pathway was significantly enriched (*p* < 0.05), with an enrichment factor of around 0.04. The enrichment observed in both the Control–Model and Model–High comparisons suggests that the JAK–STAT signaling pathway is dysregulated under pathological conditions and is responsive to therapeutic interventions. The Model–High comparison also revealed enrichment in the MAPK signaling and antigen processing and presentation pathways, implying a multi-pathway regulatory effect of the treatment.

Gene Set Enrichment Analysis (GSEA) was performed to validate the KEGG enrichment results and evaluate pathway activity at the gene set level, with a focus on the JAK–STAT signaling pathway (KEGG ID hsa04630). As shown in Figure 7C, it was significantly enriched in both the “Control vs. Model” and “Model vs. High” comparisons. The normalized enrichment score (NES) was 1.37 (*p* < 0.001) for the “Control vs. Model” comparison and 1.3 (*p* < 0.001) for the “Model vs. High” comparison, indicating a positive association between the gene set and phenotypic changes. The enrichment score (ES) curve reached its maximum at the top of the ranked gene list, and core genes (represented by red bars) were mainly distributed along the leading edge. This pattern verifies the coordinated regulation of JAK-STAT pathway genes and supports the hypothesis that this pathway is functionally relevant in both disease-modeling and therapeutic contexts.

### 2.4. Molecular Profiling of Antioxidant and Survival-Associated Signaling Pathways in Relation to GEP-2 Therapy

#### 2.4.1. Modulation of JAK/STAT Signaling Pathway, Antioxidant Response, and Apoptosis-Related Gene Transcription Levels by GEP-2

**Figure 8:**
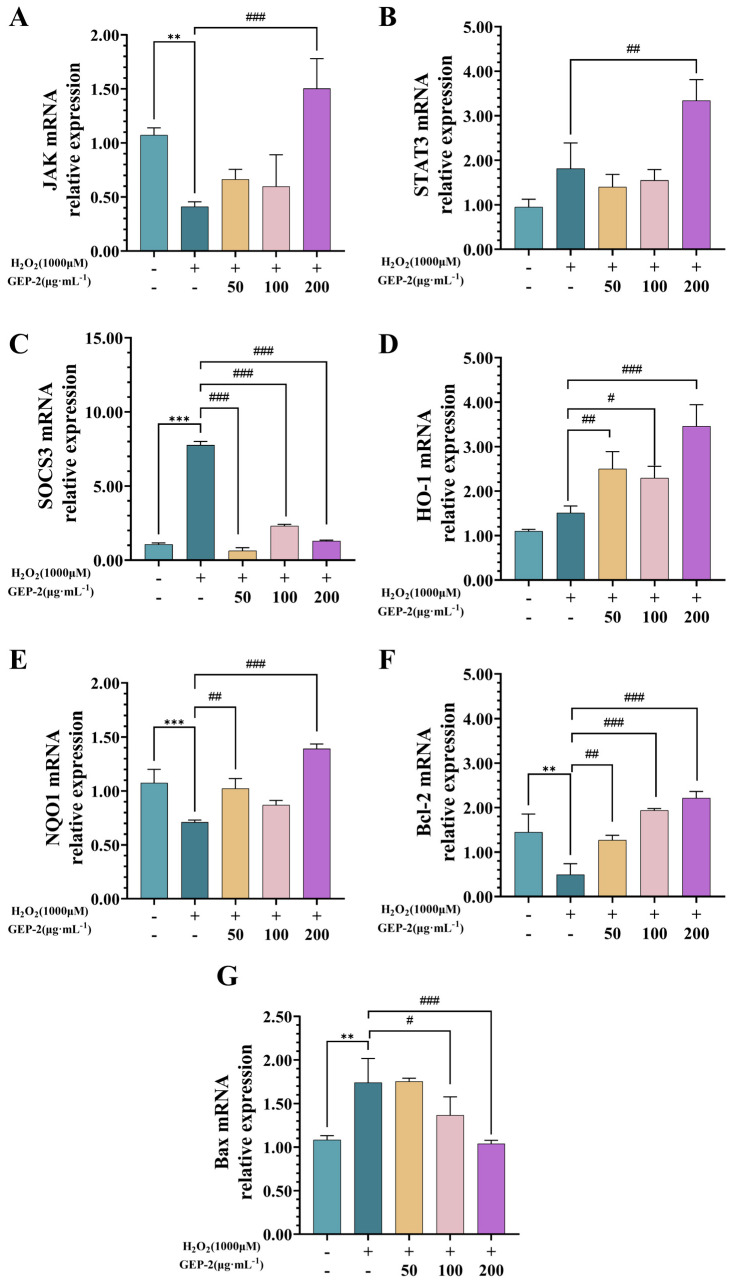
Depicts the mRNA expression levels of genes associated with the JAK/STAT pathway, apoptosis, and oxidative stress in a cellular model treated with H_2_O_2_. (**A**) JAK; (**B**) STAT3; (**C**) SOCS3; (**D**) HO-1; (**E**) NQO1; (**F**) Bcl-2; (**G**) Bax; (n = 3). Values are expressed as mean ± SEM. Compared with the Control group: ** *p* < 0.01, *** *p* < 0.001; Compared with the Model group: ^#^ *p* < 0.05, ^##^ *p* < 0.01, ^###^ *p* < 0.001.

RT-qPCR analysis was conducted to assess the mRNA expression of key constituents and downstream targets of the JAK/STAT pathway, which were identified via transcriptomic enrichment analysis. Special emphasis was placed on genes implicated in antioxidant defense and cell survival. Compared to the normal group, exposure to H_2_O_2_ significantly decreased the transcriptional levels of JAK (Figure 8A) and STAT3 (Figure 8B). This decrease was mitigated by GEP-2 pretreatment. The high-dose group exhibited a notable restoration of JAK and STAT3 mRNA expression under oxidative stress conditions. As a typical negative regulator of the JAK/STAT pathway, SOCS3 showed an increase in mRNA expression subsequent to H_2_O_2_ exposure (Figure 8C). GEP-2 pretreatment regulated SOCS3 expression in a concentration dependent manner, leading to lower SOCS3 transcript levels compared to the injury group. Consistent with these alterations, GEP-2 pretreatment was associated with elevated transcription of antioxidant-related genes, including HO-1 (Figure 8D) and NQO1 (Figure 8E). Moreover, genes related to cell survival were also affected, as manifested by increased Bcl-2 expression (Figure 8F) and decreased Bax expression (Figure 8G), and these changes were more prominent at higher concentrations. In conclusion, these results suggest that GEP-2 affects the transcriptional regulation of JAK/STAT pathway components, antioxidant defense genes, and apoptosis-related factors in H_2_O_2_-treated NCM460 cells.

#### 2.4.2. GEP-2 Augments Key Protein Expression in Nrf2 and JAK/STAT Pathways

**Figure 9:**
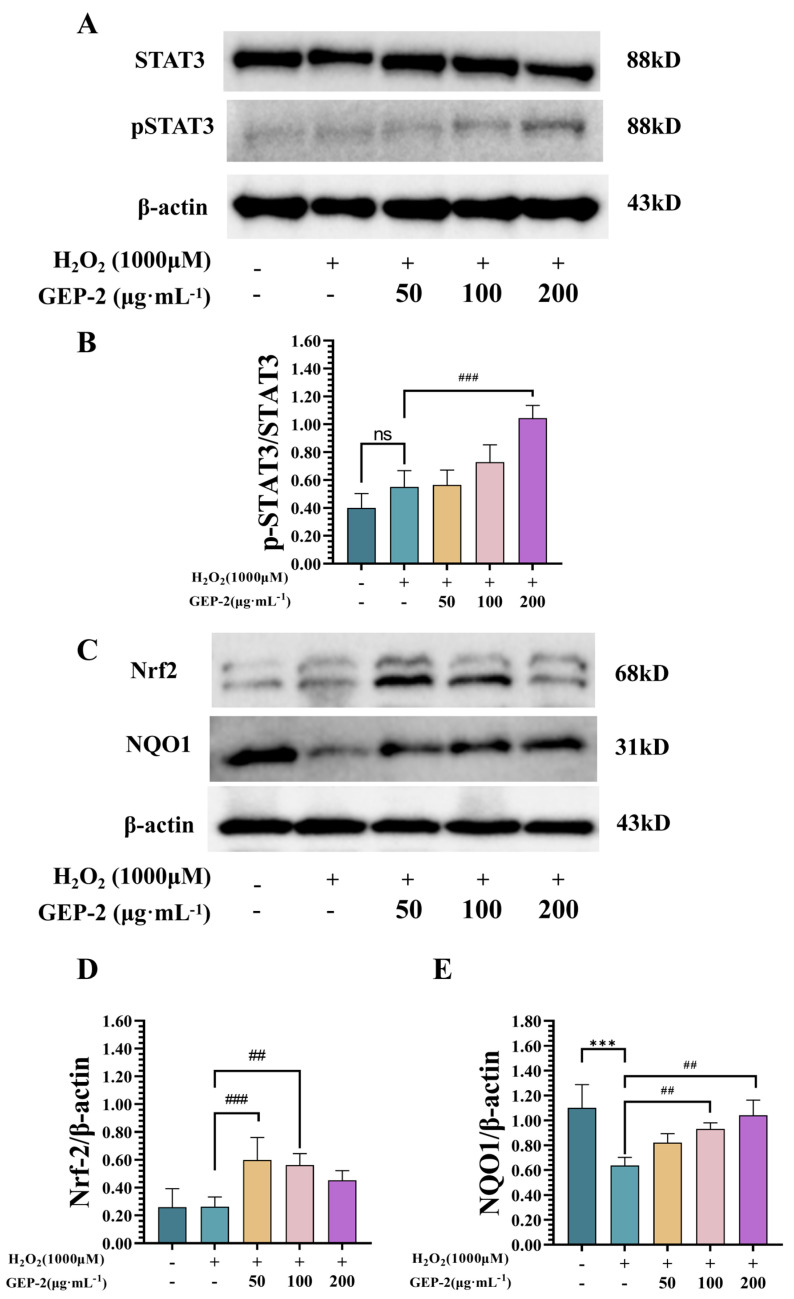
Expression levels of GEP-2 on STAT3, p-STAT3, Nrf2, and NQO1 in H_2_O_2_-treated NCM460 cells. (**A**) Representative Western blot showing the expression of STAT3, pSTAT3, and β-actin (n = 4). (**B**) Quantification of pSTAT3/STAT3 expression ratio. GEP-2 treatment (50, 100, and 200 µg·mL^−1^) significantly increased pSTAT3 levels in H_2_O_2_-induced oxidative stress compared to the untreated group. (**C**) Representative Western blot showing the expression of Nrf2, NQO1, and β-actin. (**D**) Quantification of Nrf2/β-actin expression ratio. GEP-2 treatment at 100 and 200 µg·mL^−1^) significantly enhanced Nrf2 expression compared to the untreated group. (**E**) Quantification of NQO1/β-actin expression ratio. GEP-2 treatment at 100 and 200 µg·mL^−1^ significantly increased NQO1 expression in response to oxidative stress. Values are expressed as mean ± SEM. Compared with the Control group: *** *p* < 0.001; Compared with the Model group: ^##^ *p* < 0.01, ^###^ *p* < 0.001.

To further corroborate the transcriptomic enrichment outcomes, the protein expression of pivotal components within the Nrf2 and JAK/STAT signaling pathways was analyzed. As depicted (Figure 9), the protein levels of the antioxidant transcription factor Nrf2 and its downstream target NQO1 were significantly lower in the H_2_O_2_-only group compared to the normal group (*p* < 0.01). Pretreatment with GEP-2 partially restored these levels in a dose-dependent manner, leading to significant increases in Nrf2 and NQO1 protein expression (*p* < 0.05, *p* < 0.01). These changes correspond to enhanced endogenous antioxidant defense mechanisms.

Treatment with GEP-2 was associated with the activation of the JAK–STAT pathway. In the H_2_O_2_-induced group, STAT3 phosphorylation (pSTAT3) remained at a relatively low level. Conversely, GEP-2 intervention led to a significant, concentration-dependent increase in pSTAT3 levels (*p* < 0.05), with no evident changes in total STAT3 protein expression across the groups. Significantly, the observed enhancement of STAT3 phosphorylation is consistent with the transcriptomic GSEA results, which indicated significant enrichment of the JAK–STAT signaling pathway in the High-dose group (NES = 1.37). This consistency supports a close association between the experimental protein-level findings and the pathway-level transcriptomic analysis.

## 3. Discussion

Oxidative stress plays a crucial role in the initiation and progression of intestinal diseases [23,24]. In this study, we established an oxidative stress model in NCM460 cells using hydrogen peroxide to evaluate the protective effect of GEP-2. Through a comprehensive set of research methods, we conducted a detailed analysis of the response of GEP-2 treatment to oxidative stress at different levels.

**Figure 10:**
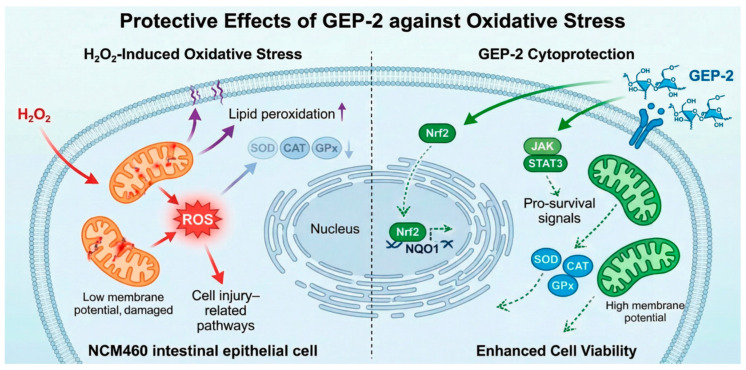
Underlying Protective Mechanisms of GEP-2 Against H_2_O_2_-Induced Oxidative Stress in NCM460 Cells. Solid arrows indicate pathways supported by experimental data in the present study, whereas dashed arrows represent proposed or associative interactions based on correlation analysis and previous literature.

The research results indicate that the pre-treatment with GEP-2 significantly alleviated the damage caused by H_2_O_2_ to NCM460 cells. Exposure to H_2_O_2_ leads to an increase in ROS levels in the cells, enhanced lipid peroxidation, inhibition of antioxidant enzyme activity, decreased MMP, and decreased cell viability [25,26]. As shown in Figure 10, we constructed an oxidative stress injury model of NCM460 cells induced by hydrogen peroxide, and found that treatment with GEP-2 could improve cell viability, while reducing reactive oxygen species, decreasing malondialdehyde content, enhancing antioxidant enzyme activity, and increasing mitochondrial membrane potential. Given the central role of mitochondria in redox regulation and cell fate determination, these findings support the protective effect of GEP-2 in NCM460 cells by maintaining mitochondrial function and redox balance, indicating the existence of a mitochondrial-related polysaccharide adaptive response to cope with oxidative stress.

Although GEP-2 exhibited weaker radical scavenging activity than vitamin C in chemical assays, it is important to note that such assays primarily reflect direct electron-donating capacity in cell-free systems and do not fully capture the complex cellular antioxidant mechanisms. Polysaccharides like GEP-2 are generally believed to exert antioxidant effects by modulating endogenous defense pathways, rather than directly neutralizing free radicals. In line with this, our study observed the activation of Nrf2 signaling and improvements in cellular oxidative stress markers, suggesting that GEP-2 primarily functions as an indirect antioxidant through the regulation of intracellular protective systems.

Furthermore, through combined transcriptome analysis, we further discovered that pathways related to oxidative stress, inflammation, and cell survival were enriched. Among these pathways, the JAK/STAT signaling pathway seemed to be closely associated with GEP-2 treatment. At the protein level, GEP-2 enhanced the phosphorylation of STAT3 and regulated the expression of SOCS3, indicating that the activation of STAT3 was regulated under oxidative stress conditions. At the same time, GEP-2 upregulated Nrf2 and its downstream target NQO1, which is consistent with the synergistic activation of antioxidant and survival signaling pathways.

Intracellular oxidative stress is primarily characterized by the excessive accumulation of reactive oxygen species (ROS) and free radicals that overwhelm the capacity of endogenous antioxidant systems. The aberrant activation of oxidative stress is closely associated with various diseases. Notably, the Nrf2/HO-1 signaling pathway is widely recognized as a central component of the cellular antioxidant defense mechanism. Previous research has indicated that *Gastrodia elata* polysaccharides, across diverse neurological [27] and tissue injury models [28], can mitigate oxidative damage markers such as ROS and MDA by facilitating Nrf2 nuclear translocation and upregulating HO-1 and NQO1 expression. Meanwhile, oxidative stress frequently activates apoptotic pathways through the induction of mitochondrial dysfunction. Studies in murine depression models [29] and PC12 cell models [30] have demonstrated that *Gastrodia elata* polysaccharides inhibit mitochondrial-mediated apoptotic cascades by modulating the Bcl-2/Bax ratio and suppressing the activation of Caspase-3, Caspase-9, and Caspase-12. These mechanisms are in line with the preservation of MMP and the enhancement of cell survival observed in the current study.

At present, studies investigating the regulatory effects of *Gastrodia elata* polysaccharides on the JAK/STAT signaling pathway are relatively limited. However, the existing evidence suggests that GEP-2 can activate the JAK/STAT pathway under oxidative stress conditions. Notably, the JAK/STAT pathway has been reported to display distinct context-dependent behaviors across various disease models. In some kinds of inflammatory models, JAK/STAT has been confirmed as a key pathway mediating anti-inflammatory and anti-apoptotic responses [31,32,33]. In contrast, in chronic metabolic diseases [34,35], the persistent over-activation of this pathway aggravates inflammatory responses and oxidative damage. These findings indicate that JAK/STAT is not strictly a “pro-inflammatory” or “protective” pathway but rather a crucial signaling hub whose outcome is dependent on cell type and stimulus intensity. Although JAK/STAT signaling is widely recognized for its role in inflammation and pathological conditions under chronic activation, emerging evidence indicates that transient STAT3 activation can function as an adaptive survival mechanism during acute cellular stress. In intestinal epithelial cells, moderate STAT3 activation has been shown to promote epithelial regeneration, maintain barrier integrity, and enhance antioxidant defense. Therefore, the observed activation of JAK2/STAT3 in the present study is more likely to represent a protective and adaptive response rather than a pathological inflammatory signal.

Integrating the results from neurological, intestinal, and tissue injury models, the cytoprotective effect of *Gastrodia elata* polysaccharides in the crucial pathological cascade of “oxidative stress–mitochondrial damage–apoptosis initiation” is not attributed to a single molecular target but is realized through the coordinated regulation of redox homeostasis, mitochondrial function, inflammatory signaling, and cell survival pathways. This study further indicates that JAK/STAT may be a previously under-recognized regulatory pathway modulated by *Gastrodia elata* polysaccharides, and the crosstalk between it and canonical antioxidant signaling pathways demands further clarification. These findings provide novel mechanistic perspectives on the molecular actions of GEP-2 in intestinal oxidative stress-related injury.

Nevertheless, this study has several limitations. Firstly, the in vitro NCM460 model is unable to fully replicate the complex intestinal environment; thus, validation in organoid or animal models is required. Secondly, although transcriptomic analysis implicated the JAK/STAT pathway, the causal relationship of GEP-2 in protection necessitates further exploration. Thirdly, the active components of GEP-2 have not been comprehensively characterized, highlighting the necessity for structure–activity relationship studies. Lastly, this study did not address the potential impacts of gastrointestinal digestion or microbial metabolism, emphasizing the significance of investigating the metabolic fate and bioactivity of GEP-2 in vivo.

However, this study has some limitations. Firstly, the present study was conducted in a single human intestinal epithelial cell line (NCM460), which may limit the generalizability of the findings. The in vitro NCM460 cell model also does not fully mimic the complex intestinal environment, so further validation using organoid or animal models is needed. Future studies involving additional intestinal epithelial models, such as Caco-2 or HT-29 cells, as well as primary intestinal epithelial cells, are warranted to further validate the protective effects of GEP-2. Secondly, while transcriptomic analysis suggested the involvement of the JAK/STAT pathway, the exact role of GEP-2 in protection still requires further investigation. To confirm the causal relationship between these pathways, loss-of-function experiments, such as siRNA-mediated knockdown of Nrf2 or STAT3, are necessary. These experiments will be addressed in future studies. Thirdly, the active components of GEP-2 have not been fully characterized, highlighting the need for structure–activity relationship studies. Additionally, this study did not account for the potential effects of gastrointestinal digestion or microbial metabolism, underscoring the necessity to assess the metabolic fate and bioactivity of GEP-2 in vivo. In the present study, GEP-2 was administered as a pretreatment prior to oxidative stress exposure, primarily reflecting a preventive protective effect. Future studies are warranted to investigate whether GEP-2 also exerts therapeutic benefits when administered after oxidative injury, thereby enhancing its translational relevance. Finally, we assessed the anti-apoptotic effect of GEP-2 by monitoring Bcl-2/Bax expression and mitochondrial membrane potential (MMP), which reflect mitochondrial-dependent apoptotic signaling. However, these methods provide indirect evidence. Future studies will incorporate more direct apoptosis assays, such as Annexin V/PI flow cytometry and caspase activity measurements, to further validate the findings.

In summary, this study demonstrates that GEP-2 can help NCM460 cells against oxidative stress by regulating mitochondrial function, antioxidant defenses, and survival-related signaling pathways.

## 4. Materials and Methods

### 4.1. Drugs and Main Reagents

H_2_O_2_ (C17736142) was sourced from Macklin Biochemical Co., Ltd. (Shanghai, China). Assay kits for MDA (G4300), T-AOC (G4313), T-SOD (G4306), CAT (G4307), and GSH (G4310), 5 × SDS protein loading buffer (PE0020), primary/secondary antibody stripping buffer (P0025), fetal bovine serum (BL205B), PBS (C8136), 0.25% trypsin–EDTA solution (C8019), Cell Counting Kit-8 (C8022), ROS detection kit (S0033S), and JC-1 assay kit (C2006), RIPA lysis buffer (G2002), PMSF (G2008-1 mL), phosphatase inhibitor cocktail (G2007-1 mL), and 50 × protease inhibitor cocktail (G2006-250 μL) were also provided by Servicebio Technology Co., Ltd. (Wuhan, China). DMEM (6125525) came from Gibco (Grand Island, NY, USA). The bicinchoninic acid (BCA) protein assay kit (BB-3401), the Super RT one-step reverse transcription premix (BL1020B) and universal SYBR Green qPCR kit (BL1304A) were purchased from Beibo Biotechnology Co., Ltd. (Shanghai, China). The 10% PAGE precast color gel kit (BL1466A) and ECL substrate (BL520B) were used, sourced from Beijing Lanjieke Technology Co., Ltd. (Beijing, China). Additionally, the DPPH free radical scavenging assay kit (C9021-100T-PKG) and Total Antioxidant Capacity Assay Kit (ABTS, Microplate Method, C8315-100T-PKG) were obtained from Shanghai Titan Technology Co., Ltd. (Shanghai, China).

### 4.2. Experimental Instruments

These included a full wavelength microplate reader and micropipettes procured from Thermo Fisher Scientific (Waltham, MA, USA), a vertical electrophoresis system and a real-time fluorescence quantitative PCR system sourced from Bio-Rad (Hercules, CA, USA), a wet transfer electrophoresis apparatus and a chemiluminescence imaging system obtained from Tanon Science & Technology (Shanghai, China), a horizontal shaker acquired from Beijing Liuyi Biotechnology (Beijing, China), an ultrapure water system provided by Merck Millipore (Darmstadt, Germany), and a gradient PCR thermal cycler supplied by Thermo Fisher Scientific.

### 4.3. Preparation of GEP-2

*Gastrodiae Rhizoma* was harvested in October 2019 from a standardized cultivation site in Chengkou County, Chongqing, China. The plant was identified as the dried rhizome of *Gastrodia elata* Blume by Longyun Li, a Research Fellow at the Chongqing Academy of Chinese Materia Medica. Polysaccharide extraction was carried out according to the protocol established by our research group [20]. Polysaccharides were prepared via a water extraction–ethanol precipitation strategy previously developed by our research group. During the pretreatment process, non-polar components and proteins were removed. Subsequently, membrane-based purification was employed to eliminate insoluble materials and low-molecular-weight constituents. The resultant extract was concentrated and dried under reduced pressure to obtain *Gastrodia elata* polysaccharide-2 (GEP-2). GEP-2 was stored at −20 °C.

The phenol–sulfuric acid method, using anhydrous glucose as the standard, was utilized to evaluate the purity of GEP-2. The obtained regression equation was y = 2.9562x + 0.0304 (r = 0.999). Based on this equation, the polysaccharide purity was calculated to be 92.89%.

GEP-2 used in the present study is the same polysaccharide fraction previously isolated and structurally characterized by our research group. In that study, GEP-2 was found to have a broad molecular weight distribution (Mn = 2.54 kDa, Mw = 9.24 kDa), with glucose as the main component (89.08%) and smaller amounts of galacturonic acid, galactose, arabinose, and rhamnose. It is a branched polysaccharide with a backbone of α-D-glucose and branches including galacturonic acid, galactose, rhamnose, and arabinose, as confirmed by NMR and methylation analysis.

### 4.4. Antioxidant Capacity Measurement

The free radical scavenging ability of GEP-2 was evaluated using DPPH and ABTS^+^ detection systems. According to the manufacturer’s protocol, solutions of GEP-2 at different concentrations were prepared and analyzed using commercially available kits. All measurements were repeated three times, and the scavenging activity was calculated based on the absorbance values.

### 4.5. Cell Culture

The NCM460 cell line was obtained from the New Drug Research and Development Laboratory at Guangzhou University of Chinese Medicine. Cell culture operations were carried out at the Analytical Center of Chongqing Academy of Chinese Materia Medica. Cells were cultivated in complete medium and incubated at 37 °C in a 5% CO_2_ humidified environment. Cells were cultured until they reached 80–90% confluence, and those in the log-phase with optimal morphology were selected for experiments.

### 4.6. Assessment of Cell Viability via the CCK-8 Assay

NCM460 cells were inoculated at an appropriate density in 96-well plates to guarantee stable adhesion. After complete attachment, the cells were exposed to GEP-2 solutions with concentrations spanning from 12.5 to 400 μg·mL^−1^ for 24 h at 37 °C. Cell viability was assessed using the CCK-8 assay. After treatment, the culture medium was replaced with serum-free medium containing CCK-8 reagent and incubated in the dark. Absorbance was measured at 450 nm, and relative cell viability was calculated based on optical density values.

### 4.7. Assessment of NCM460 Cell Viability Following GEP-2 Pretreatment Under Oxidative Stress

To establish an oxidative stress model, NCM460 cells were treated with varying concentrations of H_2_O_2_ for different time periods. Cell viability was assessed to identify conditions that caused oxidative damage while ensuring sufficient cell survival. Optimal parameters for the model were then selected.

In subsequent experiments, cells were pretreated with GEP-2 before exposure to H_2_O_2_ under these conditions. Cell viability was measured to evaluate the protective effects of GEP-2 and determine the appropriate concentration range for further analysis.

### 4.8. Cell Culture and Experimental Grouping

Cells were randomly allocated into the following groups: Control, Model (1000 μM H_2_O_2_), Low-dose (50 μg·mL^−1^), Middle-dose (100 μg·mL^−1^), and High-dose (200 μg·mL^−1^). Except for the Control group, which was cultured under standard conditions, other groups were pretreated with their respective drug concentrations for 24 h. Following this, all groups were exposed to 1000 μM H_2_O_2_ for 4 h to induce oxidative stress under consistent conditions.

### 4.9. Assessment of Intracellular ROS via DCFH-DA Staining

Following the manufacturer’s protocol, the intracellular ROS level was measured using DCFH-DA fluorescent probing after the specified treatments. Cells were incubated and stained with DCFH-DA working solution in a light-protected environment. Excess probe was removed prior to imaging, and fluorescence signals were captured using an inverted fluorescence microscope. ROS generation was quantified by measuring relative fluorescence intensity, which indicated the intracellular oxidative status.

### 4.10. Assessment of MMP Through JC-1 Staining

Following the specified treatments and manufacturer’s protocol, MMP was assessed using JC-1 fluorescent staining. Cells were incubated with JC-1 working solution in a light-protected environment. Excess dye was removed before imaging, and fluorescence signals were captured using an inverted fluorescence microscope. It was evaluated by measuring the red-to-green fluorescence intensity ratio.

### 4.11. To Quantify the Intracellular Activities of T-SOD, T-AOC, GSH, and CAT Enzymes, as Well as the MDA Content

After treatment, the cells were harvested and homogenized in 500 µL of PBS under ice-bath conditions. The cell lysate was centrifuged at 4 °C and 10,000× *g* for 10–15 min. Subsequently, the supernatant was collected and kept on ice for subsequent analysis. The supernatant was divided, with part used to determine total protein concentration, and the rest processed according to the kit instructions to measure intracellular MDA, T-SOD, T-AOC, CAT, and GSH levels.

### 4.12. Total RNA Extraction and Transcriptome Sequencing Data Processing

Following treatment, RNA was extracted using the TRIzol method according to the manufacturer’s instructions. The purity and integrity of RNA were assessed using an ultra-micro spectrophotometer and a bioanalyzer, respectively. Samples with an A260/A280 ratio ranging from 1.8 to 2.0 were regarded as pure, and an RNA integrity number (RIN) of 7.0 or higher was regarded as acceptable.

Transcriptome sequencing libraries were prepared using the Illumina^®^ Stranded mRNA protocol, which involved mRNA enrichment, fragmentation, cDNA synthesis, adaptor ligation, and PCR amplification. High-throughput sequencing was performed by PERSONALBIO Technology Co., Ltd. (Shanghai, China). The raw sequencing data were processed on the PERSONALBIO cloud platform (https://www.genescloud.cn/, accessed on 19 January 2026), including quality control, read alignment, expression quantification, and differential gene expression analysis. Differentially expressed genes were identified based on specified fold-change and adjusted *p*-value thresholds.

### 4.13. RNA Sequencing and Bioinformatic Analysis

Total RNA was extracted and subjected to high-throughput sequencing. Libraries were constructed and sequenced on an Illumina platform. Each group included three independent biological replicates (n = 3).

Clean reads were obtained after quality control filtering. The average sequencing depth per sample was approximately 25–30 million reads. The Q30 percentage exceeded 92%, and mapping rates to the reference genome were above 95%.

Differential expression analysis was performed using DESeq2 (version numberversion number 1.50.0). Genes with |log2 fold change| ≥ 1 and adjusted *p*-value (FDR) < 0.05 were considered significantly differentially expressed. *p*-values were adjusted using the Benjamini–Hochberg method.

### 4.14. Real-Time Fluorescence Quantitative PCR Detection

Total RNA was extracted from cells using the TRIzol reagent (Invitrogen Corporation, Carlsbad, CA, USA), and complementary DNA was synthesized with a commercially available reverse transcription kit for subsequent real-time quantitative PCR analysis. The relative mRNA expression levels were determined using the 2^−ΔΔCt^ method. Primer sequences were designed and synthesized by Shanghai Bogu Biotechnology Co., Ltd. (Shanghai, China), and detailed information is presented in Table 1.

### 4.15. Detection of Cellular Protein Expression via Western Blotting

Western blotting was carried out as previously described [20]. The antibodies employed were as follows: anti-Nrf2 (1:2000, B2321, Santa Cruz Biotechnology, Dallas, TX, USA), anti-NQO1 (1:2000, AF7614; Beyotime Biotechnology, Shanghai, China), anti-STAT3 (1:2000, D3Z2G), anti-phospho-STAT3 (1:2000, Tyr705; Cell Signaling Technology, Danvers, MA, USA), and anti-β-actin (1:5000, 66009-1-Ig; Proteintech, Wuhan, China). The expression of the target proteins was normalized to that of β-actin, and protein quantification was performed using ImageJ software (version number 1.8.0).

### 4.16. Statistical Methods

ImageJ software was used for image processing and statistical analysis, while quantitative data were analyzed and visualized using GraphPad Prism v9.5.0. Data are presented as the mean ± standard deviation. Pairwise comparisons between groups were performed using unpaired Student’s *t*-test, and multiple group comparisons were conducted using one-way ANOVA followed by Tukey’s post hoc test. Normality of the data was assessed using the Shapiro–Wilk test. A significance level of α = 0.05 was set, and *p* < 0.05 was considered statistically significant. All experiments were conducted with at least three independent biological replicates (n ≥ 3).

## Figures and Tables

**Table 1:** Experimental Protocols Incorporating Primer Sequence Data.

Gene	Primer	Sequence (5′ to 3′)
*JAK*	Forward	AGTGACCCTCACTTCCTGCTGT
	Reverse	GGCTGAACCAAGGATGATGTGG
*STAT3*	Forward	CTTTGAGACCGAGGTGTATCACC
	Reverse	GGTCAGCATGTTGTACCACAGG
*SOCS3*	Forward	CATCTCTGTCGGAAGACCGTCA
	Reverse	GCATCGTACTGGTCCAGGAACT
*HO-1*	Forward	CCAGGCAGAGAATGCTGAGTTC
	Reverse	AAGACTGGGCTCTCCTTGTTGC
*NQO1*	Forward	CCTGCCATTCTGAAAGGCTGGT
	Reverse	GTGGTGATGGAAAGCACTGCCT
*Bax*	Forward	TCAGGATGCGTCCACCAAGAAG
	Reverse	TGTGTCCACGGCGGCAATCATC
*Bcl2*	Forward	ATCGCCCTGTGGATGACTGAGT
	Reverse	GCCAGGAGAAATCAAACAGAGGC
*GAPDH*	Forward	GTCTCCTCTGACTTCAACAGCG
	Reverse	ACCACCCTGTTGCTGTAGCCAA

## Data Availability

The raw data supporting the conclusions of this article will be made available by the authors on request.
